# Effect of transcutaneous auricular vagal nerve stimulation on the fatigue syndrome in patients with gastrointestinal cancers — FATIVA: a randomized, placebo-controlled pilot study protocol

**DOI:** 10.1186/s40814-023-01289-z

**Published:** 2023-04-22

**Authors:** Mortimer Gierthmuehlen, Nadine Höffken, Nina Timmesfeld, Kirsten Schmieder, Anke Reinacher-Schick

**Affiliations:** 1 Department of Neurosurgery, University Medical Center Knappschaftskrankenhaus Bochum, In Der Schornau 23-25, 44892 Bochum, Germany; 2 Department of Hematology, Oncology and Palliative Medicine, University Medical Center St. Josef-Hospital Bochum, Gudrunstrasse 56, 44791 Bochum, Germany; 3 Department of Medical Informatics, Biometry and Epidemiology, Ruhr-University Bochum, Universitaetsstrasse 150, 44801 Bochum, Germany

**Keywords:** Cancer-related fatigue, Vagal nerve stimulation, Cytokines

## Abstract

**Background:**

Cancer-related fatigue (CRF) is defined as a “distressing, persistent, subjective sense of physical, emotional, and/or cognitive tiredness or exhaustion related to cancer or cancer treatment that is not proportional to recent activity and interferes with usual functioning.” CRF is frequently observed in cancer patients even before the initiation of tumor therapy. Its cause is not clear, but in addition to primary effects of therapy, a tumor-induced elevated level of inflammatory cytokines may play a role. Transcutaneous auricular vagal nerve stimulation (taVNS) is a noninvasive way to activate central nervous pathways and modulate pain perception and the immune system. It has positive effects on autoimmune conditions and can also improve fatigue associated with Sjogren’s syndrome. It is the main purpose of this feasibility study to investigate the feasibility of daily taVNS against CRF. Therefore, the stimulation protocol of the newly introduced smartphone app of the manufacturer is evaluated. Additionally, the effect taVNS on CRF and quality of life (QoL) shall be evaluated.

**Methods:**

Thirty adult patients with gastrointestinal tumors during or after treatment, relevant CRF (Hornheide questionnaire) and life expectancy > 1 year, are enrolled. Patients are randomized to treatment or sham arm and be informed that they will either feel the stimulation or not. Treatment group will receive left-sided tragus above-threshold stimulation with 25 Hz, 250 µs pulse width, and 28-s/32-s on/off paradigm for 4 h throughout the day for 4 weeks. Sham group will receive no stimulation via a nonfunctional electrode. A daily stimulation protocol with time and average intensity is automatically created by a smartphone app connected to the stimulator via Bluetooth®. Multidimensional Fatigue Inventory-20, Short-Form 36 and Beck Depression Inventory questionnaires will be filled out before and after 4 weeks of stimulation.

**Discussion:**

Primarily, the patients’ daily stimulation time and intensity will be evaluated through the electronic protocol after 4 weeks. Secondarily, the effect of taVNS on cancer-related fatigue and QoL will be measured through the questionnaires. As taVNS seems to modulate inflammatory cytokines, this noninvasive method may — if accepted by the patients — be a promising adjunct in the treatment of cancer-related fatigue.

**Trial registration:**

The study was approved by local ethics committee (21–7395) and registered at the DRKS database (DRKS00027481).

## Introduction

### Background

Fatigue is one of the most common short- and long-term side effects of cancer therapy. The National Comprehensive Cancer Network (NCCN) guidelines from 2015 define cancer-related fatigue (CRF) as “distressing, persistent, subjective sense of physical, emotional, and/or cognitive tiredness or exhaustion related to cancer or cancer treatment that is not proportional to recent activity and interferes with usual functioning” [1]. At the time of cancer diagnosis, up to 40% of patients complain of fatigue symptoms [2]. In the further course of therapy, more than 90% of patients suffer from cancer-related fatigue (CRF), which can be pronounced on a physical, emotional, and cognitive level. Common symptoms are tiredness, lack of strength and drive, concentration disorders, and a decreased quality of life (QoL), often associated with severe impairments in the private and professional performance. Fatigue can occur before, during, or after cancer treatment and can last for different lengths of time [1]. The 2-question test according to Whooles has become an accepted method to differentiate CRF from an underlying depressive disorder [3]. Even after the specific oncological therapy, up to 40% of all cancer patients continue to suffer from CRF, which greatly impairs their QoL [4]. The cause of CRF is multifactorial and not entirely elucidated yet. Therefore, a validated treatment concept does not yet exist. However, a chronic inflammatory process and an associated increase in inflammatory cytokines seem to play a role in the development of CRF [5]. In addition to their pro-inflammatory effects, both radiation and chemotherapy might have direct influence on the central nervous system and could contribute to CRF as well. Chemotherapy directly influences oligodendrocytes and neuronal stem cells and can therefore cause demyelination and vascular damage, especially in the context of neuroinflammation. One meta-analysis found a positive correlation between the severity of CRF and the concentration of inflammatory cytokine [6]. Thus, chronic inflammation appears to play an important role in the development of CRF.

A disbalance of the autonomic nervous system in favor of the sympathetic nervous system also seems to play a role [7, 8]. In this context, a dysregulation in the hypothalamic circuit [9], an alteration in the serotonergic system [10], and a disturbance in the sleep–wake cycle [11] could contribute to the development of CRF.

Invasive vagus nerve stimulation (iVNS) has been approved for the treatment of epilepsy since the late 1990s and depression since the early 2000s [12]. Its noninvasive variant, the transcutaneous auricular vagus nerve stimulation (taVNS), has been available for a few years and is based on the anatomical observation that one part of the ear — the tragus — is exclusively innervated by sensory fibers of the vagus nerve. Electrical stimulation of this area leads to activation of the brain comparable to the iVNS [13, 14]. taVNS has only few side effects, most notably skin irritation [15]. Via the “anti-inflammatory reflex loop,” taVNS leads to an anti-inflammatory effect, a reduction in inflammatory cytokines [16–18], and modulates cardiac vagal tone [19]. In the context of the current corona pandemic, it has also been observed in Covid-19 patients that taVNS reduces the inflammatory cytokine interleukin (IL) 6 [20]. In 2018, De Couck described the protective influence of the N. vagus on the prognosis of cancer patients [21] and encouraged the scientific society to perform further studies on the VNS in oncological patients [22]. This idea was taken up by Tibensky et al. in 2020, who discussed potential positive effects of the parasympathetic nervous system certain types of cancer (e.g., breast cancer), while prostate cancer, for example, may in theory be negatively influenced by an increased level of acetylcholine [23]. Furthermore, it has been observed that taVNS leads to an increase in heart rate variability (HRV) [24, 25] in patients with tinnitus. This observation may be important for cancer patients, since a reduced HRV is a predictive value for a poorer outcome [26, 27] and appears to be associated with CRF [7]. In summary, the stimulation of the vagal nerve has probably no positive effect on cancer patients by optimizing the actual cytostatic therapy. Instead, VNS has the potential to improve the quality of life — which has a significant impact on the survival of cancer patients — by reducing fatigue as a cancer- and cytostatic-specific side effect.

### Objectives

Based on the abovementioned observations that CRF might be caused by inflammatory processes which may be modulated by taVNS, it is the aim of this feasibility study to investigate whether daily taVNS is accepted by the patients as a treatment against CRF during this 4-week trial. Additionally, we will evaluate whether CRF can be influenced by left-sided taVNS. Established and sensitive questionnaires for depression, fatigue, und QoL will be used: Short-Form 36 (SF-36), Beck Depression Inventory (BDI), and Multidimensional Fatigue Inventory (MFI20). We chose this combination of questionnaires with respect to both the sensitivity for changes during taVNS and to the length and number of questions.

The SF36 questionnaire is established in many diseases. It is commonly applied in patients with fatigue [28, 29], autoimmune diseases [30, 31], Parkinson’s [32], and cancer [33–36]. It comprises 36 questions focusing on general aspects of QoL and will be applied to investigate the general wellbeing of our patients before and after taVNS. BDI examines the existence of depression and has already proven to respond to iVNS in epilepsy patients [37], to cervical noninvasive VNS in migraine patients [38], and to taVNS in patients with depression [39]. MFI20 is an established questionnaire in the assessment of cancer-related fatigue [40, 41]. Additionally, baseline values for a general population [42] and minimal clinically important differences are available [43, 44].

### Aim

It is the aim of the study to investigate whether 4 h of daily taVNS is accepted as a treatment against CRF and whether the symptoms of CRF and QoL can be influenced by taVNS in patients with gastrointestinal tumors and clinically apparent CRF.

- Primary endpoint: Acceptance of and compliance to taVNS, measured with the mean duration of taVNS per day. taVNS, is considered accepted when at least 80% of patients use the stimulator an average of at least 2 h per day.
- Secondary endpoint: Effect of taVNS on clinical scores (MFI20, BDI, and SF-36)

## Methods

The study will be performed at the University Medical Center Knappschaftskrankenhaus Bochum in cooperation with the University Medical Center St. Josef-Hospital, Bochum. Both hospitals belong to the University Medical Center of the Ruhr-University Bochum, Germany. The Department of Hematology, Oncology, and Palliative Medicine of the St. Josef-Hospital will identify, inform, and recruit the patients in its outpatient clinic and will transfer them to the Department of Neurosurgery at the Knappschaftskrankenhaus Bochum.

The attached SPIRIT table (Table 1) explains when the interventions and questionnaires will be performed within the study during enrolment, visit 0, and visit 1 (Table 1: Schedule of enrollment, intervention, and assessments).

**Table 1:**
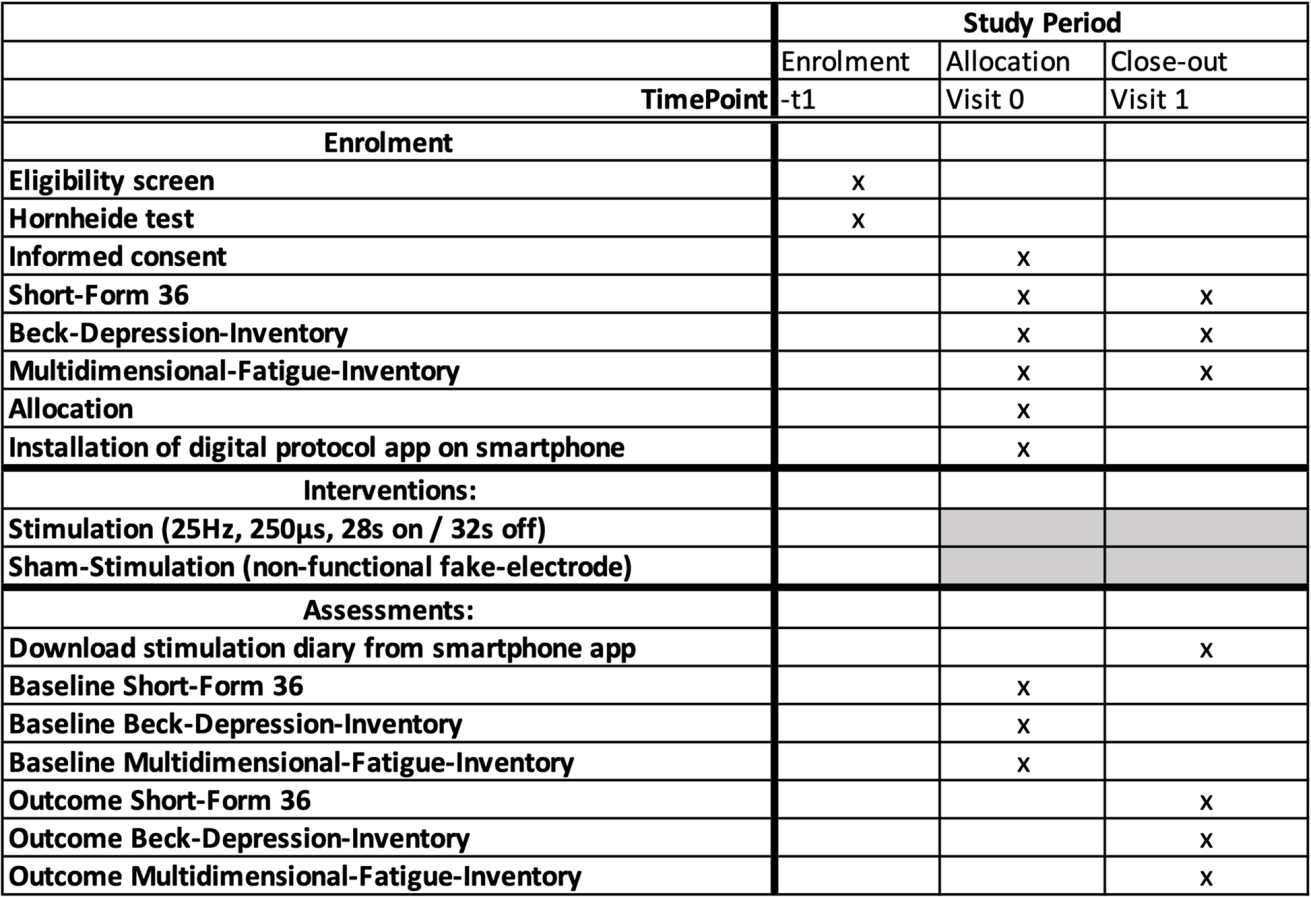
Schedule of enrollment, intervention, and assessments

### Patients

In accordance with the NCCN guidelines, all admitted patients of the Department of Haematology, Oncology and Palliative Medicine at the St. Josef-Hospital Bochum, who suffer from malignant gastrointestinal tumors, are routinely screened with a short CRF screening test (Hornheide’s questionnaire). If — before the initiation of an adjuvant therapy, a severity grade > 4 and a disability score > 5 are detected, eligible patients will be asked to participate in the study.

GDPR-compliant data collection will be pseudonymized in the eCRF of the university’s REDCap® system. The patients are automatically randomized by REDCap® into the stimulation or sham group (*n* = 15 each). Since the patients in the sham group will also be instructed in the use of the stimulator (which describes a tingling sensation of the stimulation in the ear), sham patients are told that they will receive a stimulation below the perception threshold. Since the rule “the more, the better” is not necessarily applicable in vagal nerve stimulation and a stimulation below the perception threshold could theoretically be effective, this procedure seems to be justifiable.

#### Inclusion criteria

- Patients during or after therapy of a gastrointestinal tumor
- Life expectancy > 1 year
- Clinically relevant CRF (Hornheide’s questionnaire)
- Age > 18 years old
- No or stable depression for at least 4 weeks

#### Exclusion criteria

- Severe psychiatric disease (e.g., schizophrenia)
-  > 8 scores on NGASR suicidal questionnaire
- Ongoing vagal nerve stimulation or s.p. vagotomy
- Relevant cardiac disease, e.g., bradycardic arrhythmia, insufficiency, and s.p. infarction
- Active implant, e.g.. ICD, pacemaker, neurostimulator, cochlear implant, and VP shunt
- Inability to understand the study
- Progressive neurological disease (e.g., Parkinson’s, epilepsy, multiple sclerosis)
- Pregnancy
- Prostate carcinoma
- Skin disease like infection or eczema at the stimulation site
- Anatomical anomaly at the stimulation site
- Other severe condition preventing the successful participation in the study

#### Abort criteria

- Occurrence of an exclusion criteria
- Occurrence of severe cardiac arrhythmias

### Data logging

The patient is asked to download the manufacturer’s app available for Android and iOS, which connects to the stimulator via Bluetooth® and logs the stimulation time and average stimulation intensity per day. A cloud connection or a registration is not necessary. The app also shows the patient when the recommended total stimulation time of 4 h/day has been reached. Although the stimulator switches off after 4 h of continuous stimulation, there is no automatic blocking of the stimulator after 4 h of cumulative stimulation. This does not pose a problem as — according to the exclusion criteria — only patients who understand the study protocol will take part in the study. After 4 weeks, the respective data is exported from the patient’s smartphone and will be analyzed with respect to the mean daily stimulation time and mean daily stimulation intensity.

### Questionnaires

We will use 3 well-established questionnaires investigating QoL, depression, and fatigue: Short-Form 36 (SF-36, Beck Depression Inventory (BDI), and Multidimensional Fatigue Inventory (MFI20). These questions are digitally stored in the REDCap® system and will be filled out by our study nurses together with the patients. If a patient is unable to physically appear to the second visit, he/she will be given a temporary patient’s access to the respective REDCap® eCRF in order to fill out the missing questionnaires.

### Stimulation group

The device we use in this trial is the tVNS-L system manufactured by tVNS GmbH, Erlangen, Germany. It has a CE certificate for left-sided transcutaneous auricular vagal nerve stimulation with a fixed frequency of 25 Hz, pulse width of 250 µs, and 28-s on/32-s off protocol, and a variable stimulation intensity. It is an over-the-counter available system which can be bought in the manufacturer’s online shop. The stimulation will be performed at the tragus of the left ear in accordance with the CE certificate. The stimulation intensity is adjusted by the patient just above the perception threshold, until a light tingling is felt at the electrode. The stimulation shall be done for in total 4 h throughout the day, but not necessarily for 4 consecutive hours.

### Sham group

For stimulation in the sham group, the manufacturer offers a non-connected fake electrode which looks identical to the real electrode. Resistors within the electrode report a sufficient impedance to the stimulator so the control-LED of tVNS-L will always show good contact to the skin. The patient will be told that he/she is either in the group where he can feel the stimulation or in the group where the stimulation is below the threshold. The patients will therefore be blinded, the doctors won’t.

#### Study plan

As explained in the SPIRIT table (Table 1), all patients with gastrointestinal tumors are routinely screened for CRF with the Hornheider’s questionnaire. If CRF is detected (severity grade > 4 and a disability score > 5) and the patient could be eligible to participate (inclusion/exclusion criteria), he/she is informed about the study and receives a study flyer. If the patient wants to participate, he/she gets an appointment at the outpatient clinic of the Neurosurgical Department of the University Medical Center Knappschaftskrankenhaus Bochum.

### Visit 0

At this time, the patient will give his informed consent. Then he/she receives the stimulator and is instructed how to use it. He/she is also asked to install the manufacturer’s app on the smartphone (“tvns patient” on Google Play® and Apple App Store®). The stimulator is paired with the smartphone app, logs the stimulation intensity and duration for each day, and reminds the patient to stimulate. The data of the app can only be read by the patient and will be documented by the physician at the end of the study, together with the patient.

Personal data like age, sex, height, and weight will be recorded. Also, it will be noted whether the patient is still under radio- or chemotherapy. The three questionnaires are filled out, and the patient is discharged. After 1 week, he/she will be called by our study nurse and asked if he/she has any questions or problems with the stimulation. The last visit will be 4 weeks later. The patients are advised to contact the principal investigator whenever a side effects occur.

### 4-week-stimulation phase

#### Visit 1

After 4 weeks, the patient has a new appointment at the outpatient clinic of the Neurosurgical Department. The questionnaires are filled out, and the stimulation statistics are read from the smartphone app, under the patient’s supervision. The patient’s weight is measured again. If a patient is unable to physically appear to the second visit, he/she will be given a temporary patient’s access to the respective REDCap® eCRF to fill out the missing questionnaires. The stimulator is given back to the hospital.

### Justification of sample size

Acceptance and compliance are considered successful when at least 80% of patients fulfills the acceptance criteria as defined in the primary endpoint. A total of 30 patients will be recruited, 15 in each of the two groups “sham” and “stimulation.” The sample size of 15 subjects per group was based on feasibility, as taVNS against cancer-related fatigue has not been investigated before. It is also based on studies investigating non-invasive VNS against sleep deprivation [45], lupus [46], and Sjoegren syndrome [47]. The choosen sample size adheres to the recommendations of sample sizes in feasibility studies [48, 49]. We will probably see a power of approximately 80% to detect if less than 50% of the patients fulfills the acceptance criteria with an exact one-sided binomial test at significance level 5%. Furthermore, with this sample size, the expected length of the 95% confidence interval for the adherence rate within one group lies between 0.38 and 0.44 and between 0.28 and 0.33 for the pooled rate. The expected length was calculated using the Agresti-Coull method and assuming a true rate between 50 and 80%.

### Statistical analysis

For the descriptive analyses of the primary endpoint and all binary secondary endpoints absolute numbers, percentages and 95% confidence interval (CI) using Agresti-Coull method will be calculated. For comparisons between groups, odds ratio and corresponding 95% CI will be calculated. For continuous secondary endpoints mean, standard deviation and the 95% CI for the mean or median and quartiles will be calculated. For comparisons, difference between means and corresponding 95% CI or difference in location parameters using the Hodge-Lehman method and corresponding 95% CI will be determined. Furthermore, for the primary endpoint, we will test the null hypothesis “acceptance rate is higher than 80%” with an exact one-sided binomial test with significance level 5%.

## Discussion

### Endpoints

The main objective and primary endpoint of the presented feasibility study protocol are to investigate the adherence of patients to daily stimulation of the left ear during daily routine. A positive adherence will be stated if at least 80% of the participants use the stimulation at least 50% of the recommended time (4 h) per day. The second generation of in-ear electrodes for the tVNS-L system provides a fixation ring which is put around the earlobe to provide better stability compared to the NEMOS® system. A recent study with NEMOS® reported a high drop-out rate due to practical reasons [50], and to our knowledge, our study will be the first to systematically investigate adherence to the protocol with the new electrode. Furthermore, it will be the first to evaluate therapy adherence by using the newly developed and published smartphone apps which provide an automatic stimulation diary. Last, patients suffering from fatigue are harder to motivate to participate in studies. It therefore will be of great interest if they will adhere to a daily stimulation protocol of 4 h over a period of 4 weeks.

Our findings will give us the information needed for a future randomized control trial, such as recruitment rates, inclusion/exclusion criteria, adherence to the therapy, and sample size. It will also provide insight on how oncological patients suffering from fatigue can handle a daily stimulation protocol and which questionnaires will be the most sensitive for the investigation of taVNS in a CRF population. To this end, the secondary endpoints are the respective questionnaires for fatigue and QoL.

### Regulatory considerations

In May 2021, the EU regulation (EU) 2017/745 was implemented in the German law under the so-called MPDG and replaced the old MPG. Ever since, the approval of a study with a non-CE-marked medical device by the ethics committee has become much more challenging. Therefore, to perform this study under the §47(3) of the German MPDG (comparable with the old §23b MPG), the stimulator had to be CE certified. Within this type of study (CE-marked device and no further invasive procedure like blood drawing), no study insurance would have been necessary. However, we chose a road insurance and a study insurance for all participants to reasonable costs.

The manufacturer tVNS GmbH offers the tVNS system in two different versions: L-Version and R-Version (Research). The tVNS-L features a fixed set of parameters (25 Hz, 250 µs, 28 s on/32 s off) with a variable intensity. It has a CE mark for the treatment of inflammation, Crohn’s disease, epilepsy, and other conditions (https://shop.t-vns.com/?lang=en). tVNS-R instead consists of the same technical device, driven by another firmware, which allows also variable settings of frequency and pulse width. The R version therefore does not possess a CE mark. tVNS-R would have allowed us to treat the sham group with a different frequency than 25 Hz, e.g., 1 Hz. Although tVNS-R with its variable settings would have been more interesting for us as a research group, the abovementioned limitations made it almost impossible to perform this study with tVNS-R.

This study was then approved by the local ethics committee of the Ruhr-University Bochum under the registration number 21–7395. Several studies investigated taVNS in healthy volunteers, e.g., in behavioral research [25, 51–53], and none of them reported relevant side effects. In their review, Redgrave et al. summarized the results of over 1300 participants and reported skin irritation (16.7%), headache (3.3%), nasopharyngitis (1.7%), and vertigo (1.1%) as the most frequently seen side effects. Only in 2.6% of the patients had to be withdrawn from the study due to side effects [54]. Even when performing chronic taVNS, i.e., lasting several weeks, only a few side effects were found [55, 56]. In oncological patients, a positive effect of taVNS is postulated [21–23]. The investigation of taVNS in patients with cancer-related fatigue therefore seems to be justifiable.

### Sham intervention

The research version of tVNS (tVNS-R) with a variable set of stimulation parameters would have been scientifically more interesting, especially when it came to defining the sham group. However, the abovementioned legal issues encouraged us to use the CE-marked version tVNS-L with a fixed set of parameters and only variable stimulation intensity.

Defining the control/sham group has been discussed in literature. While some suggest stimulation of entirely different body areas [57], others chose the earlobe, which is not innervated by the vagal nerve, for control stimulation [58]. However, stimulation of the earlobe or other parts of the ear might cause interfering muscle activation [59, 60]. Interested and informed patients could also easily identify that they are in the control group by inserting the electrode “in a wrong” way. Therefore, we chose a fake electrode offered by the manufacturer with no electric connection between the stimulator and the patient.

In case the results indicate that a larger study could be feasible, we propose that future studies could consist of three groups of participants: the first group with a sham electrode, the second group with subthreshold stimulation, and the third with above-threshold stimulation. If there is no difference in the sub- and above-threshold stimulation (i.e., both show a similar effect), future studies could be limited to sham stimulation vs. subthreshold stimulation, therefore allowing a true blinded, even double-blinded, setting.

Another way to noninvasively stimulate the vagal nerve is via transcutaneous stimulation at the neck level. This device is called Gammacore® and delivers current through two skin electrodes. Sham stimulation can be performed by applying a fake stimulator which only delivers mechanical vibration instead of electric current. This method is used in a study which investigates transcutaneous VNS at the neck level against fatigue in lupus patients (NCT05315739). Another study investigates taVNS in a heterogenous group of patients with different types of cancer. The sham intervention is not exactly specified (NCT04563013).

### Data logging on smartphone

In the latest consensus paper, data logging was recommended to monitor the patients’ adherence to the stimulation therapy [61]. Patients are encouraged to install the official app of the manufacturer on their smartphone which can be paired with the stimulator via Bluetooth®. The app does not require cloud function, and the data stays on the patient’s smartphone and has recently been introduced. If a patient does not wish to install the app, he/she is asked to write a paper-based diary of the daily stimulation periods. In this case, stimulation intensity could not be logged.

### Data protection

All data will be recorded in a pseudonymized database. The REDCap® system will not record the patient’s name but only his/her number. The link between number and patient name will only be done in a table stored on a different clinical network storage in our hospital. The rules of GDPR also apply for this study.

### Data monitoring

Data monitoring will be performed by the Department of Medical Informatics, Biometry and Epidemiology, Ruhr-University Bochum, which runs and supervises the REDCap ® system used in this study.

## Data Availability

As this is a report of our trial protocol, no data is available yet.

## Abbreviations

BDI: Beck depression inventory
CRF: Cancer-related fatigue
GDPR: General data protection regulation
HRV: Heart rate variability
ICD: Implantable cardiac defibrillator
iVNS: Invasive vagal nerve stimulation
MFI20: Multidimensional fatigue inventory 20
MPG: Medizin Produkt Gesetz (old German Medicinal Devices Act)
MPDG: Medizinprodukterecht-Durchführungsgesetz (new German Medicinal Devices Act)
NGASR: Nurses’ global assessment of suicide risk
SF36: Short-form 36
taVNS: Transcutaneous auricular vagal nerve stimulation
VNS: Vagal nerve stimulation
VP shunt: Ventriculoperitoneal shunt
QoL: Quality of life
