# Correction: Effect of transcutaneous auricular vagal nerve stimulation on the fatigue syndrome in patients with gastrointestinal cancers — FATIVA: a randomized, placebo‑controlled pilot study protocol

**DOI:** 10.1186/s40814-023-01331-0

**Published:** 2023-06-03

**Authors:** Mortimer Gierthmuehlen, Nadine Höffken, Nina Timmesfeld, Kirsten Schmieder, Anke Reinacher‑Schick

**Affiliations:** 1 Department of Neurosurgery, University Medical Center Knappschaftskrankenhaus Bochum, In Der Schornau 23‑25, 44892 Bochum, Germany; 2 Department of Hematology, Oncology and Palliative Medicine, University Medical Center St. Josef-Hospital Bochum, Gudrunstrasse 56, 44791 Bochum, Germany; 3 Department of Medical Informatics, Biometry and Epidemiology, Ruhr-University Bochum, Universitaetsstrasse 150, 44801 Bochum, Germany

**Correction:** **Pilot Feasibility Stud 9, 66 (2023)**

**https://doi.org/10.1186/s40814-023-01289-z**

Following publication of the original article [1], the authors identified an error to the presentation of the author names. The given names and family names for all authors were erroneously transposed.

The author group has been updated above and the original article [1] has been corrected.
